# High-Temperature Tolerance in Multi-Scale Cermet Solar-Selective Absorbing Coatings Prepared by Laser Cladding

**DOI:** 10.3390/ma11061037

**Published:** 2018-06-19

**Authors:** Xuming Pang, Qian Wei, Jianxin Zhou, Huiyang Ma

**Affiliations:** School of Mechanical and Power Engineering, Nanjing Tech University, Nanjing 211816, China; 15722929056@163.com (Q.W.); jxzh1973@163.com (J.Z.); mhy52361554@163.com (H.M.)

**Keywords:** solar-selective coating, multi-scale cermets, laser cladding technology, long-term thermal stability

## Abstract

In order to achieve cermet-based solar absorber coatings with long-term thermal stability at high temperatures, a novel single-layer, multi-scale TiC-Ni/Mo cermet coating was first prepared using laser cladding technology in atmosphere. The results show that the optical properties of the cermet coatings using laser cladding were much better than the preplaced coating. In addition, the thermal stability of the optical properties for the laser cladding coating were excellent after annealing at 650 °C for 200 h. The solar absorptance and thermal emittance of multi-scale cermet coating were 85% and 4.7% at 650 °C. The results show that multi-scale cermet materials are more suitable for solar-selective absorbing coating. In addition, laser cladding is a new technology that can be used for the preparation of spectrally-selective coatings.

## 1. Introduction

Solar absorbers have been widely used for photo thermal conversion because of their high energy-conversion efficiency and environmentally-friendly energy storage functionality [1,2,3]. Currently, thermal conversion has been commercially carried out in concentrating solar power (CSP) systems, in which the solar receivers are one of the key parts to dominate the entire performance of CSP. In order to increase the efficiency of the power generation system, it requires heat transfer fluid (HTF) with materials of high photo-thermal conversion at high temperatures of ≥600 °C [4]. As a key component of concentrating CSP, cermet coatings consisting of highly infra-red (IR)-transparent and solar absorbing materials, such as Pt/Al_2_O_3_, Co/WC, W/AlN, and Mo/Si_3_N_4_ have been used to develop solar selective absorber coatings for medium–high temperature photo-thermal conversion applications, because of their excellent absorptance (α) in the wavelength range of 0.3 μm < λ < 2.5 μm and low thermal emittance (ε) in the IR region (2.5 μm < λ < 25 μm) [5,6,7,8,9,10,11,12]. At present, typical cermet based solar selective absorber coatings have a multilayer structure. In order to achieve excellent absorption, the thickness of each layer must be controlled based on the optical interference principle of the multiple layers. For conventional multi-layer selective absorber coatings, the absorption is about 90%, and the emission is less than 10% based on the previous documents. In addition, solar absorptance of 80% and thermal emittance (<10%) are desired properties for optimum single layer coatings [10]. However, the optical performance of the multilayer coatings could degrade with coupled influences of high temperature and air, due to component aggregation, oxidation, diffusion, and change in microstructure when the coatings are used for long hours at high temperatures. Owing to these limitations, it is required to develop new solar absorber coatings with excellent thermal stability for future high-temperature applications (>600 °C).

As solar selective coatings, TiN-based cermets exhibit many attractive properties, including a high α in the solar spectral range, and a low ε in the deep IR region [8]. Titanium carbide (TiC) also exhibits interesting features, such as good oxidation resistance, electrical conductivity, and high thermal stability. Particularly, the oxidation resistance of TiC is higher than that of TiN. Therefore, the thermal stability of TiC-based cermet coatings is better than that of TiN-based cermets. In addition, it is noteworthy that single-layer coatings of multi-scale tungsten, consisting of nano- and micro-particles, have been fabricated on stainless steel substrates with a solar absorptance of 83% and a thermal emittance of 11.6% at 300 K [12]. Therefore, multi-scale cermet-based solar selective absorber coatings are expected to exhibit improved photo-thermal conversion performance and thermal stability.

At present, several methods such as electrochemical plating, plasma spraying, hydrothermal methods, and magnetron sputtering have been used to prepare spectrally selective solar absorber coatings with various structures. Magnetron sputtering is the main technique used in industrial production, but it requires a vacuum environment for preparation of the coating. The laser cladding method has been widely used for the preparation of protective, hard, and wear-resistant coatings [13]. Unlike magnetron sputtering, this technique does not require a vacuum environment, making it suitable for industrial production. However, studies on preparation of solar selective absorber coatings by the laser cladding method have not been reported so far.

In the present work, a single-layer, multi-scale TiC-Mo/Ni cermet coating was fabricated using the laser cladding technique, and its microstructure and optical performance were investigated.

## 2. Experimental

Micro- (~2 μm) and nano- (~50 nm) particles of Ni (99.5% pure), Mo (99.5% pure), and TiC (~2 μm, 99.5% pure) are shown in Table 1. Commercially pure Ni, Mo, and TiC powders in different weight ratios (Table 1) were put into an organic solvent mixture of propanetriol, ethanediol, and isopropyl alcohol. The mixed powders were sprayed onto 316Ti stainless steel substrates (30 mm × 30 mm × 1 mm) using compressed air as a carrier gas in order to preplace the coating. Then, the preplaced coatings (samples 1 and 2) were heated to 200 °C, resulting in the evaporation of the organic solvent. Finally, the coating (sample 2) was treated using a carbon dioxide laser with the protection of argon during the laser cladding process. The process parameters were: laser power (P) = 1 kW, scanning speed (v) = 3 mm/s, and beam diameter (D) = 3 mm. For comparison, a single-scale, multi-scale TiC-Mo/Ni cermet coating (sample 3) was also prepared on a stainless steel substrate. 

The reflectance spectra R(λ) of the cermet coatings were measured in the wavelength range of 0.3–2.5 μm using a Lambda 950 UV spectrophotometer. The near-normal spectral reflectance in the wavelength range of 2.5–25 μm was measured using a Thermo Scientific Nicolet iS10 spectrometer (Thermo Fisher Scientific, Waltham, MA, USA). The α and the ε of the coatings can be calculated using the following equations [14].

(1) $$\alpha =\frac{\int_{0.3}^{2.5}\left(1-R\left(\lambda \right)\right)I_{S}\left(\lambda \right)d\lambda }{\int_{2.5}^{25}I_{S}d\lambda }$$

(2) $$\varepsilon =\frac{\int_{2.5}^{25}\left(1-R\left(\lambda \right)\right)I_{b}\left(\lambda ,t\right)d\lambda }{\int_{2.5}^{25}I_{b}\left(\lambda ,t\right)d\lambda }$$

where *λ* is the wavelength, *R*(*λ*) is the reflectance at *λ*, *I_b_* (*λ*, *t*) is the black body spectral radiation at temperature *t*, and *I*_s_(*λ*) is the solar spectral radiation at AM = 1.5.

The structural characterization of the coatings was carried out by X-ray diffraction (XRD) employing Cu K_α_ radiation (*λ* = 1.54056 Å). The metallographic structure of the coating was observed using a 4XC metallurgical microscope (Shang Guang, Shanghai, China). The surface morphologies and chemical composition were investigated by scanning electron microscopy (SEM) (FEI Quanta 250F, FEI, Hillsboro, OR, USA). The microstructural features of the samples were analyzed by transmission electron microscopy (TEM) (JEM-100CX, JEOL, Tokyo, Japan). 

## 3. Results and Discussion

**Figure 1:**
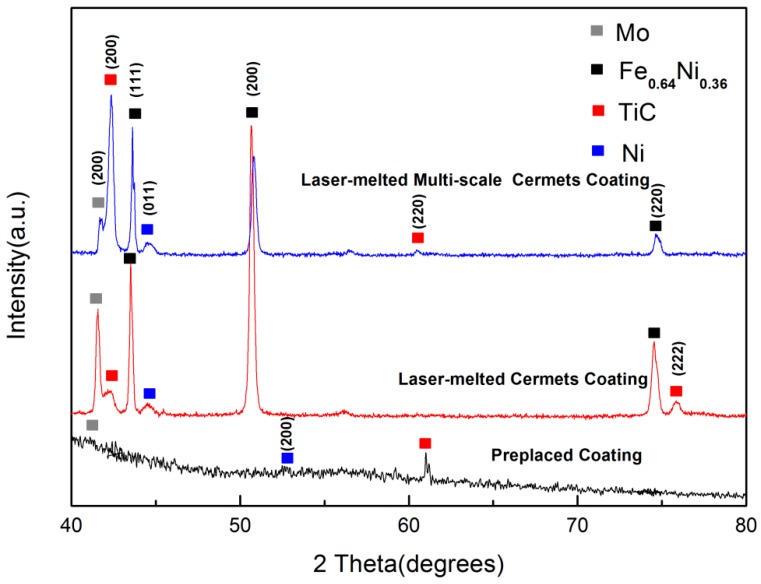
XRD patterns of the preplaced coating, cermet coating, and multi-scale cermet coating prepared by laser cladding.

The XRD patterns of the cermet coatings are shown in Figure 1. Peaks related to Mo, Ni, and TiC are observed in the XRD pattern of the preplaced coating. High-intensity diffraction peaks of Ni, Mo, and TiC appear in the XRD patterns of the laser clad coatings. Furthermore, the diffraction peaks of the Fe_0.64_Ni_0.36_ solid solutions are observed in the XRD patterns of the laser clad cermet coating and multi-scale cermet coating. The formation of the Fe_0.64_Ni_0.36_ solid solution results from the crystallization of the mixed liquid phase of Fe and Ni, owing to the laser melting of Fe and Ni based on the process characteristics of laser cladding.

**Figure 2:**
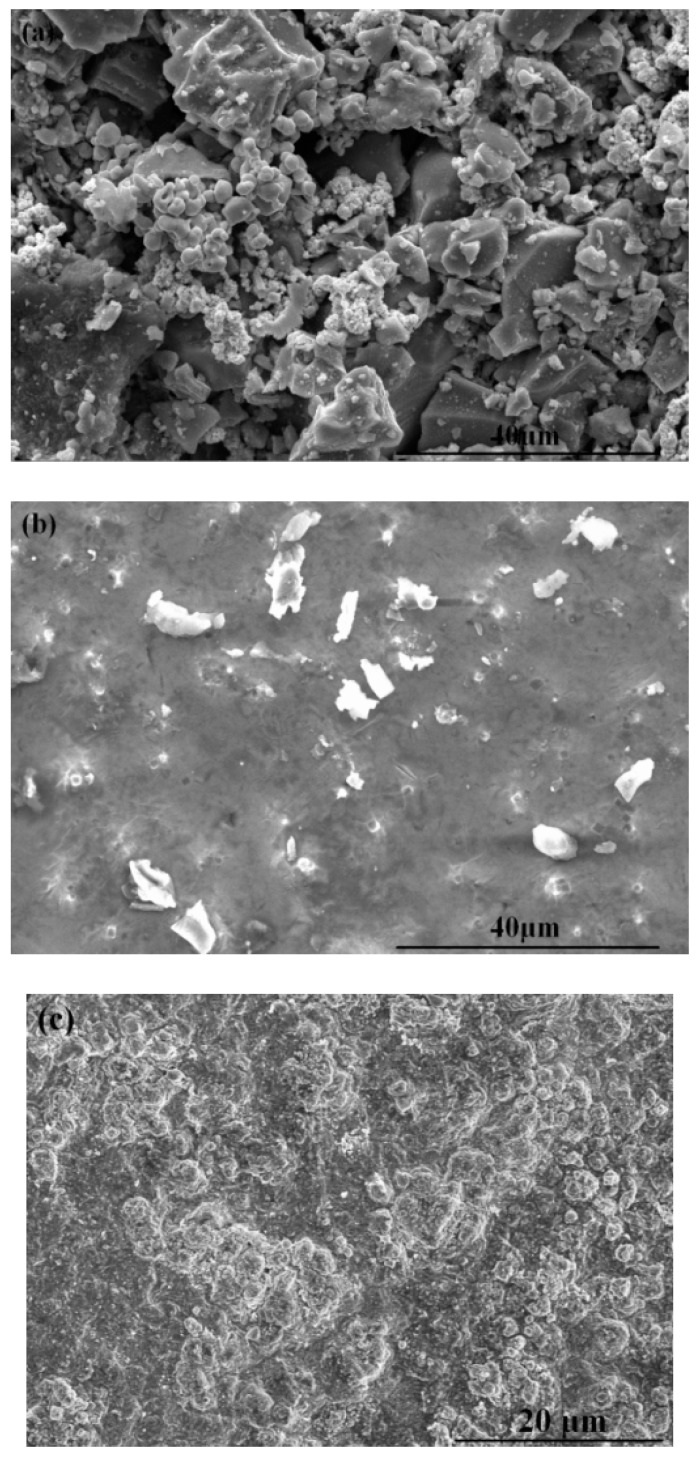
Scanning electron microscopy (SEM) micrographs of the (**a**) preplaced coating, (**b**) laser cladding cermet coating, and (**c**) multi-scale cermet coating.

The SEM micrographs of the coatings are shown in Figure 2. From Figure 2a, the particles of TiC-Ni/Mo cermet form a certain degree of agglomerate, which the preplaced coating is not compacted with pores. Furthermore, the metal powders are smaller in size compared to the large and blocky-shaped TiC powders. According to Figure 2b,c, the TiC-based cermet and the multi-scale TiC-based cermet coatings are both compacted, because of the high-energy density of laser cladding. The melting, diffusion, and crystallization of the metal and ceramic particles take place rapidly during laser processing. The surface of the multi-scale cermet coating is not smooth, which may help in increasing the optical trap absorption. A comparison of the SEM micrographs of the preplaced coating and the multi-scale cermet coating indicates that the solar absorptance of the cermet coating fabricated by laser cladding could be higher.

**Figure 3:**
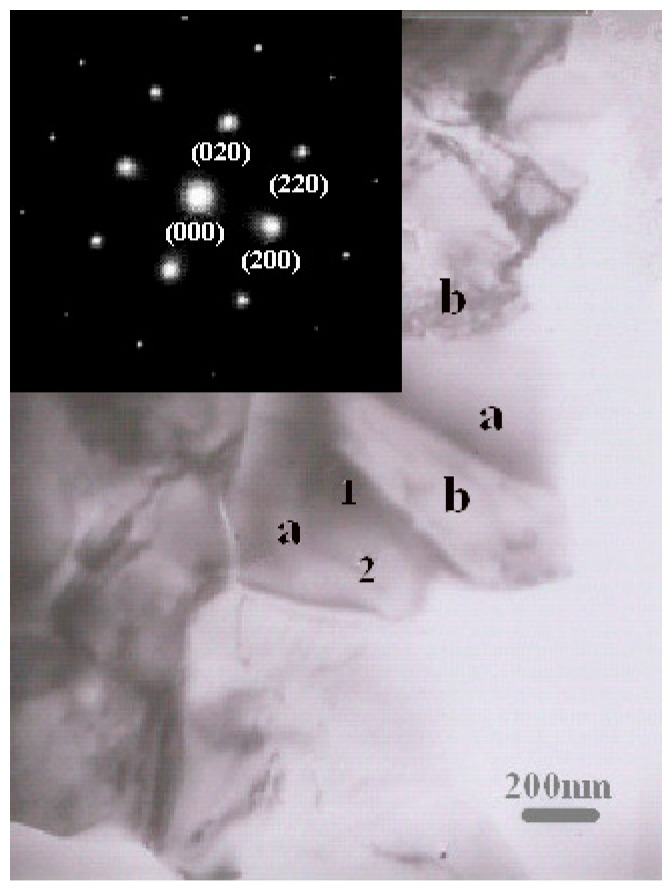
Bright-field transmission electron microscopy (TEM) images of the cermet coating prepared by laser cladding.

Figure 3 shows the bright-field TEM image of the cermet coating prepared by laser cladding. The ceramic and metal phases are evident, marked as zone a and zone b, respectively. However, the contrast of the ceramic grain is different. Because Mo diffuses to the TiC ceramic and forms (Ti, Mo) a C solid solution around the TiC [15], the content of Mo is higher near the shell of the TiC solid solution. Therefore, regions with different contrast, such as zone 1 and zone 2, are observed. The inset shows the selective area electron diffraction (SAD) pattern, in which zones 1 and 2 are selected simultaneously using a diaphragm. The TEM and the SAD results indicate that the diffraction spots and the crystal structure of zones a and b are similar. 

**Figure 4:**
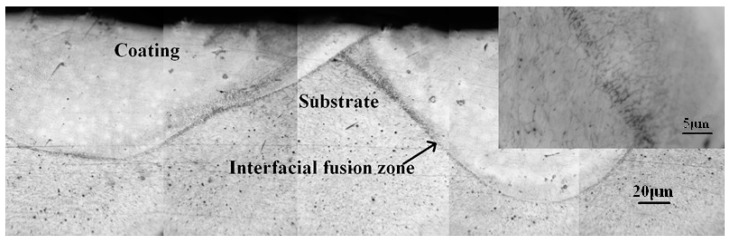
Macro-morphology of the transverse section of coating fabricated by laser cladding.

Figure 4 shows the morphology of the transverse section of the cermet coating prepared by laser cladding. The typical metallographic structures of a coating, such as coating, interfacial fusion zone, and substrate, are observed in Figure 4. The interfacial fusion zone exhibits an adendritic structure, as shown in the inset. Under laser processing, particles melt rapidly and form a welding pool on the substrate surface. A temperature gradient, perpendicular to the interface, forms because of the low temperature of the substrate and the high temperature of the welding pool. The liquid in the welding pool rapidly crystallizes along the heat-extracted direction perpendicular to the interface, which results in the formation of dendritic structures. Therefore, the laser cladding technique ensures formation of a high-quality metallurgical bonding between the coating and the substrate. These results indicate that the laser cladding method has unique advantages for the fabrication of solar selective absorber coatings.

**Figure 5:**
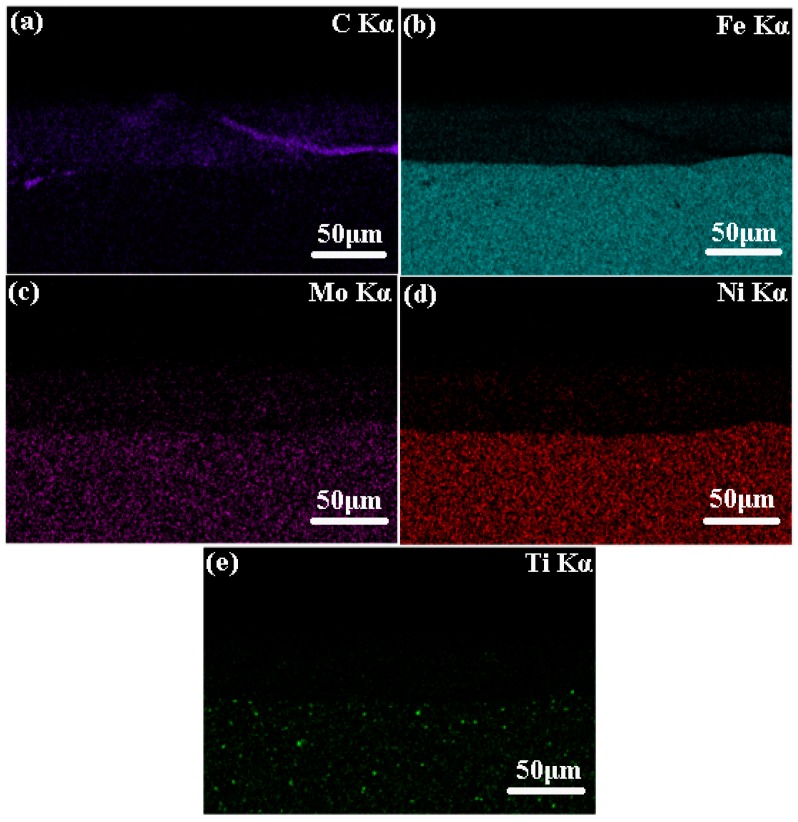
Elemental distribution analysis of the cermet coating. (**a**) distribution of C; (**b**) distribution of Fe; (**c**) distribution of Mo; (**d**) distribution of Ni; (**e**) distribution of Ti.

Figure 5 shows the elemental distribution analysis of the coating obtained by energy dispersive X-ray spectroscopy (EDS). For the TiC-Ni/Mo samples, the C element is predominant in the coating, which originates from the TiC ceramic. As mentioned before, laser processing leads to substrate surface melting according to the process characteristics of laser cladding. A small amount of Fe is also observed in the coating. The Ni, Mo, and Ti elements are detected in both the zones of the coating and the substrate because these elements coexisted in the zones.

**Figure 6:**
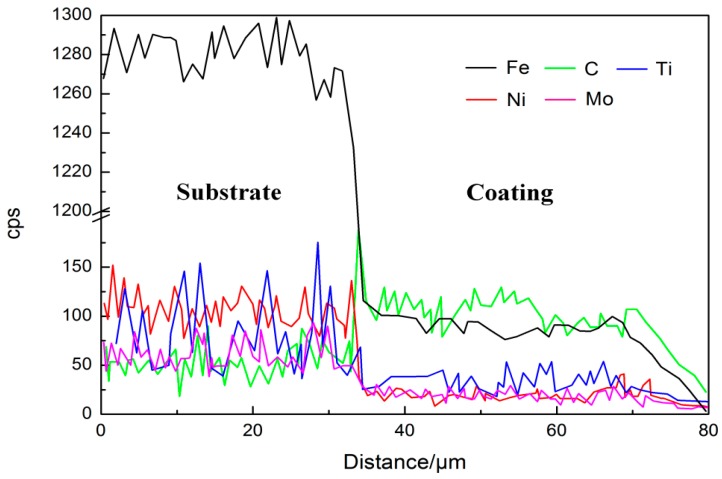
Energy dispersive X-ray spectroscopy (EDS) linear scanning profiles of the main elements across the cermet coating.

The line-scanning profile in Figure 6 shows the EDS linear scanning result of the main elements across the TiC-Ni/Mo cermet coating, demonstrating a spatially inhomogeneous distribution at the interface between the substrate and the coating. This result was further confirmed by the elemental mappings shown in Figure 5. The composition changes significantly at a distance ≥45 μm from the coating surface to the substrate. These results are in good agreement with the analyses of elemental distribution (see Figure 5).

**Figure 7:**
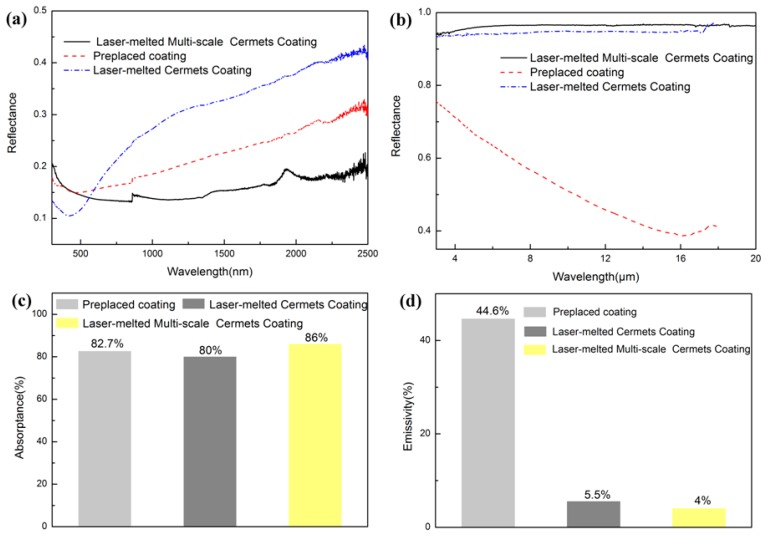
Reflectance spectra and absorption performance of the laser clad cermet coatings: (**a**) reflectance spectra in the UV to near infra-red (IR) regime, (**b**) reflectance spectra in the IR region, (**c**) absorptance of the coatings, and (**d**) emittance of the coatings.

Figure 7a shows the reflectance curves of the coating in the wavelength range of 0.3–2.5 µm. The laser clad cermet coating has a low reflectivity in the wavelength range of 0.3–0.6 µm. Then the reflectance of the laser clad cermet coating and the preplaced coating increases after 600 nm. The increased reflectance of the laser clad cermet coating also results from the high-energy density during laser cladding. The formation of compacted and continuous metallurgical bonding layer after melting and crystallization of the powders within a short time (see Figure 2) leads to a mirror effect near the IR region. Therefore, the reflectance of the cermet coating is gradually improved. It is to be noted that the reflectivity of the multi-scale cermet coating is slightly high before 0.5 µm and low in the wavelength range of 0.5–2.5 µm. The whole reflectance of the multi-scale coating is low in the ultra-violet to the near IR regime. This result indicates the improved absorption of the multi-scale cermet coating. The multi-scale nature and the presence of different kinds of metals can enlarge the absorption spectrum. The enhancement for the multi-scale coating in the solar absorptance results from the high light-trapping efficiency of nano-scale Ni/Mo particles. As can be seen from Figure 2c, it is obvious that the morphology is dense and uniform. For coarse and fine nano metal particles, the interaction with light is obviously improved, which are collections of large concentrations of nearly free electrons [2,4]. In addition, TiC exhibits high absorption at a short wavelength. Furthermore, the absorptance increases at shorter wavelengths because of the resonance effect between the ceramic and metal during laser cladding. Therefore, the improved absorptance of the coating could be attributed to the multi-scale and laser cladding. 

According to the reflectance spectra of the coatings in the IR region, as shown in Figure 7b, the surface thermal emissivity of the coatings relates to surface condition, temperature, and material. Owing to the metallic nature of the laser clad coating on the surface, the mirror effect exists in the IR region, which leads to a higher reflectance. In addition, the reflectance value is almost unchanged in the IR region, which may result from the compacted surface of the laser clad coating. Because of the presence of pores and cracks in the preplaced coating, the infrared reflectance considerably decreases with an increase in the infrared wavelength, as shown in Figure 7b.

The absorptance and the emittance of the coatings were calculated using Equations (1) and (2), and the results are shown in Figure 7c,d. The α of the preplaced coating is 82.7% and the ε is 44.6%, which is attributed to its poor microstructure. The α and the ε of the single-scale and multi-scale coatings prepared by laser cladding are 80% and 5.5%, 86% and 4%, respectively. Evidently, the photo-thermal conversion of the multi-scale coating is significantly higher.

**Figure 8:**
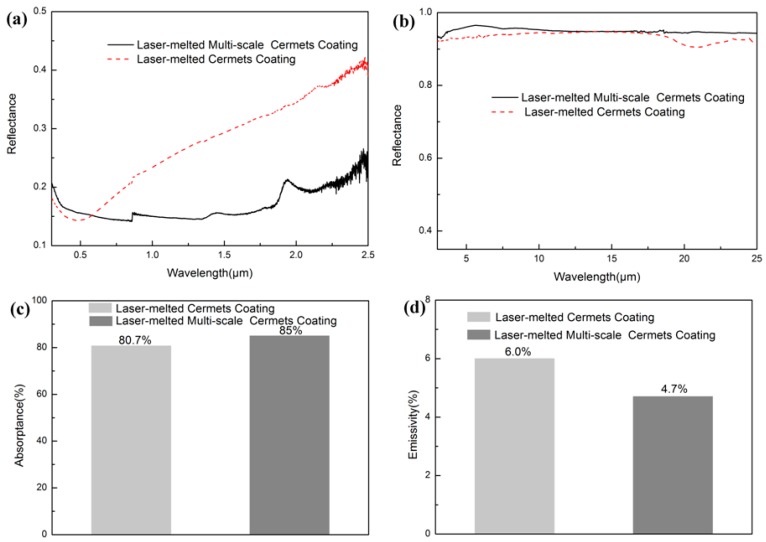
Reflectance spectra and absorption performance of the laser clad cermet coatings after heat treatment at 650 °C for 200 h: (**a**) reflectance spectra in the UV to near-IR regime, (**b**) reflectance spectra in the IR region, (**c**) absorptance of the coatings, and (**d**) emittance of the coatings.

Figure 8a shows the reflection spectra of the cermet coatings after heat treatment at 650 °C for 200 h. Compared with the coating before heat treatment, the reflectance of the solar absorbing coating after heat treatment is relatively higher in the wavelength range of 0.3–0.6 μm and lower at wavelengths >0.6 μm. When the wavelength is <12.5 μm, the reflectance of the coating in the IR region is almost unchanged, and then decreases slightly with increase in the wavelength, as evident from Figure 8b. Furthermore, the absorption of the multi-scale cermet coating is better in the wavelength range of 0.5–2.5 μm, and the emittance is lower because of higher reflectance in the IR region. The absorptance and the emittance of the single-scale and multi-scale coatings after heat treatment are 80.7 and 6%, and 85 and 4.7%, respectively, as shown in Figure 8c,d. This indicates the excellent optical stability of the multi-scale cermet coating at high temperature.

**Figure 9:**
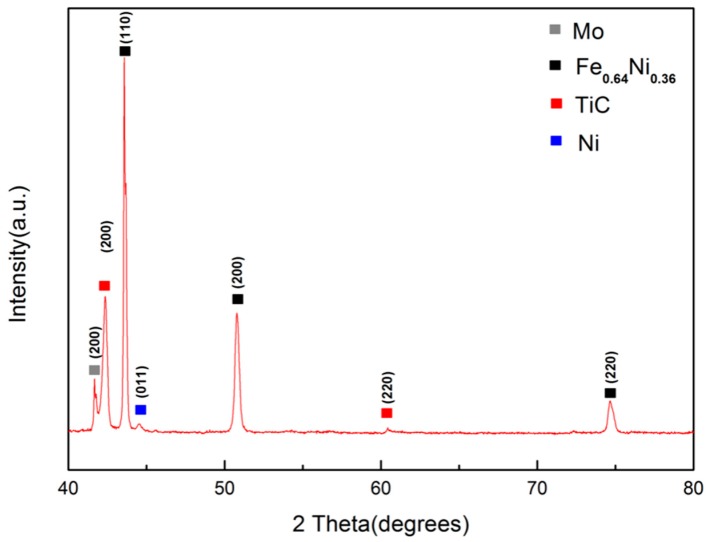
XRD pattern of the heat treated multi-scale cermet coating fabricated by laser cladding.

Figure 9 shows the XRD pattern of the multi-scale cermet coating prepared by laser cladding after heat treatment. The phase composition of the coating is unchanged after heat treatment at 650 °C for 200 h, which indicates good thermal stability. Thus, the single-layer, multi-scale TiC-based cermet coating prepared by laser cladding is suitable for high-temperature applications.

Based on a previous report [10], a solar absorptance of 80% and a thermal emittance <10% are the optimum properties of a single-layer solar absorber coating fabricated using the sputtering technique. Therefore, the laser cladding method could be a novel technology in the domain of solar absorber coatings.

## 4. Conclusions

In conclusion, a single-layer, multi-scale TiC-Ni/Mo cermet coating was fabricated using the laser cladding method under atmospheric conditions. Due to the excellent thermal stability of the coatings, the performance of cladding coating remains similar before and after anneal at 650 °C for 200 h. The results indicate that the multi-scaled features (from 50 nm to 5 µm) of the particles have helped achieve spectral selectivity and enhance the solar absorptivity from 80% to 86% of uniform micro-particles. In addition, a low thermal emissivity of 4.7% was achieved after anneal at 650 °C for 200 h in air and a high selective receiver efficiency of ~85.0% was achieved for laser cladding coatings. Multi-scale cermets are promising candidates as spectrally-selective coatings for high-temperature applications. The laser cladding method could be used in the domain of spectrally-selective coatings as a new technology.

## Figures and Tables

**Table 1:** Chemical composition of the samples.

Sample No.	Micron TiC (wt %)	Micron Ni (wt %)	Nano Ni (wt %)	Micron Mo (wt %)	Nano Mo (wt %)
1	60	30	0	10	0
2	60	30	0	10	0
3	60	15	15	5	5

1: Preplaced coating, 2: laser-melted cermet coating, 3: laser-melted, multi-scale cermet coating.
